# Sarcomatoid Variant of Hepatocellular Carcinoma: Rare and Deadly

**DOI:** 10.7759/cureus.94965

**Published:** 2025-10-20

**Authors:** Alaita Fatima Bakhtiari, Malyka Batool, Smavia Hameed, Kainat Osama, Muhammad Atique, Imran Ali Syed, Usman Iqbal Aujla

**Affiliations:** 1 Gastroenterology and Hepatology, Pakistan Kidney and Liver Institute and Research Center, Lahore, PAK; 2 Radiology, Pakistan Kidney and Liver Institute and Research Center, Lahore, PAK; 3 Histopathology, Pakistan Kidney and Liver Institute and Research Center, Lahore, PAK

**Keywords:** chemotherapy, doxorubicin, hepatitis c, immunohistochemistry, rare liver neoplasm, sarcomatoid hepatocellular carcinoma, spindle cell tumor

## Abstract

Sarcomatoid hepatocellular carcinoma (SHCC) is a rare and aggressive subtype of hepatocellular carcinoma (HCC), often associated with poor prognosis due to its late presentation, high metastatic potential, and resistance to treatment. We report the case of a 69-year-old male with newly diagnosed hepatitis C infection who presented with right hypochondrial pain, weight loss, and constitutional symptoms. Imaging revealed multiple hepatic masses, portal vein thrombosis, and extensive lymphadenopathy. Biopsy of a dominant liver lesion confirmed SHCC, with histopathological features of atypical spindle cells and immunohistochemical positivity for cytokeratin (CK), glutamine synthetase, and heat shock protein-70 (HSP-70), confirming hepatocellular lineage. Given the unresectable nature of the disease and its advanced stage at diagnosis, the patient received doxorubicin-based chemotherapy as a palliative measure; however, despite initial symptomatic improvement, the overall survival was limited to 5.5 months from the time of initial presentation. This case highlights the diagnostic and therapeutic challenges of SHCC and underscores the importance of early histological confirmation, as well as the urgent need to explore novel systemic therapies for this condition.

## Introduction

Hepatocellular carcinoma (HCC) is the most common primary liver tumor, accounting for 80% of all liver cancers [1], and is one of the leading causes of cancer-related deaths worldwide [2]. Sarcomatoid hepatocellular carcinoma (SHCC), also referred to as spindle cell HCC, is a rare histological subtype of HCC that remains poorly understood. The reported incidence of SHCC ranges from 0.79% in histologically confirmed HCC cases to 1.8% among those who underwent surgical resection [3].

SHCC tends to behave more aggressively than conventional HCC, often presenting with larger tumors, extrahepatic spread, and higher histological grades, all of which contribute to poorer clinical outcomes, reduced overall survival, and high recurrence rates [4,5]. SHCC is histologically characterized by spindle-shaped cells with elongated, oval nuclei, prominent nucleoli, and eosinophilic cytoplasm, exhibiting both epithelial and mesenchymal differentiation [6]. The pathophysiology of SHCC involves the upregulation of genes associated with epithelial-mesenchymal transition and inflammatory responses. Compared to classic HCC, sarcomatoid variants often show upregulated programmed death-ligand 1 (PD-L1) expression and are more densely infiltrated by immune cells, suggesting a more immunologically active tumor microenvironment [3].

SHCC is more commonly seen in older males, with a mean age of approximately 59 years [6]. It shares similar risk factors with conventional HCC, including chronic viral hepatitis, alcohol use, and cirrhosis, and has previously been observed to develop in association with prior anticancer treatments [7,8]. Management of SHCC remains a challenge. Despite the use of surgical resection, locoregional approaches, and systemic therapies, outcomes remain suboptimal due to poor responsiveness to treatments such as transarterial chemoembolization (TACE) and early recurrence following curative interventions [9].

To contribute to this body of knowledge, we present a case of SHCC in a 69-year-old male. This case adds to the limited literature on SHCC and highlights the importance of considering this variant in patients presenting with atypical imaging features and aggressive tumor behavior.

## Case presentation

A 69-year-old male, a farmer by occupation, presented to the hepatology clinic with a one-month history of right hypochondrial pain, radiating to the back, and partially relieved with analgesics. The abdominal pain was associated with nausea, vomiting, and constipation. He also reported an unintentional weight loss of 15 kg over the past four months, accompanied by a significant loss of appetite. He denied any history of alcohol intake and drug abuse.

He was diagnosed with hepatitis C infection two months back and had not yet received any antiviral treatment. There was no prior history of hepatic decompensation. On examination, the patient appeared frail, with a tender hepatomegaly extending below the right costal margin.

Laboratory investigations revealed normal liver function tests (total bilirubin level = 0.7 mg/dL, aspartate aminotransferase (AST) = 30 IU/L, alanine aminotransferase (ALT) = 31 IU/L, alkaline phosphatase (ALP) = 606 IU/L, and serum albumin = 3.8 g/dL). A complete blood count revealed a hemoglobin level of 10.8 g/dL, a white blood cell count of 9 × 10^3/μL, and a platelet count of 266 × 10^9/μL. Coagulation profile and renal function tests were within normal limits. Tumor markers showed a serum alpha-fetoprotein (AFP) level of 7.24 ng/mL and an elevated CA 19-9 level of 602.2 U/mL (Table 1).

**Table 1:** Laboratory Investigations mg/dL – milligrams per deciliter; IU/L – international units per liter; g/dL – grams per deciliter; ng/mL – nanograms per milliliter; U/mL – units per milliliter; μL – microliter; L – liter; mEq/L – milliequivalents per liter.

Parameter	Patient Value	Reference Range
Total Bilirubin	0.7 mg/dL	0.2-1.2 mg/dL
Alanine aminotransferase (ALT)	30 IU/L	0-55 IU/L
Aspartate aminotransferase (AST)	31 IU/L	5-34 IU/L
Alkaline phosphatase (ALP)	606 IU/L	40-130 IU/L
Serum Albumin	3.8 g/dL	3.5-5 g/dL
Alpha fetoprotein (AFP)	7.24 ng/mL	≤7.0 ng/mL
Carbohydrate antigen 19-9 (Ca 19-9)	606 U/mL	35 U/mL
Hemoglobin (Hb)	10.8 g/dL	12.3-16.6 g/dL
White cell count (WBC)	9.0 ×10³/μL	4.6-11.38 ×10³/μL
Platelets	266 ×10⁹/L	150–450 ×10⁹/L
International normalized ratio (INR)	1.14	0.7-1.5
Serum Creatinine	0.73 mg/dL	0.7-1.2 mg/dL
Serum Sodium	128 mEq/L	135-145 mEq/L
Serum Potassium	5.44 mEq/L	3.5-5.1mEq/L

**Figure 1:**
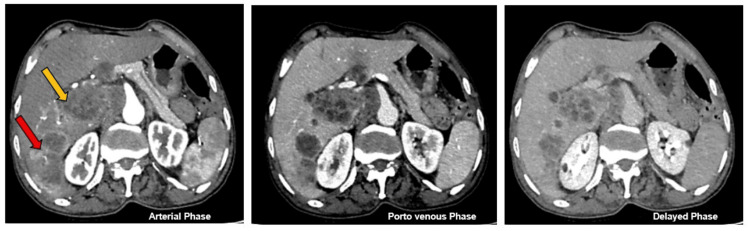
Computed tomography images of the sarcomatoid hepatocellular carcinoma. The red arrow highlights an infiltrative mass in segment VI of the liver, demonstrating heterogeneous peripheral enhancement during the arterial phase with corresponding washout on the portovenous and delayed phases. The yellow arrow indicates a large nodal conglomerate at the porta hepatis exhibiting similar enhancement features.

Triphasic abdominal computed tomography (CT) demonstrated irregular hepatic contours with multiple focal lesions, predominantly within the right lobe. The largest lesion, an infiltrative mass measuring 7.7 centimeters in segment VI, exhibited heterogeneous peripheral enhancement during the arterial phase with subsequent washout on the portovenous and delayed phases. Additionally, a large nodal conglomerate was identified at the porta hepatis, exhibiting similar enhancement characteristics (Figure 1). CT scan also revealed a tumor thrombus involving the right posterior branch of the portal vein.

Based on imaging characteristics, the differential diagnosis included atypical hepatocellular carcinoma, combined hepatocellular-cholangiocarcinoma, or extensive hepatic and nodal metastases from an unknown primary malignancy. Therefore, an ultrasound-guided biopsy of the hepatic lesion was performed.

**Figure 2:**
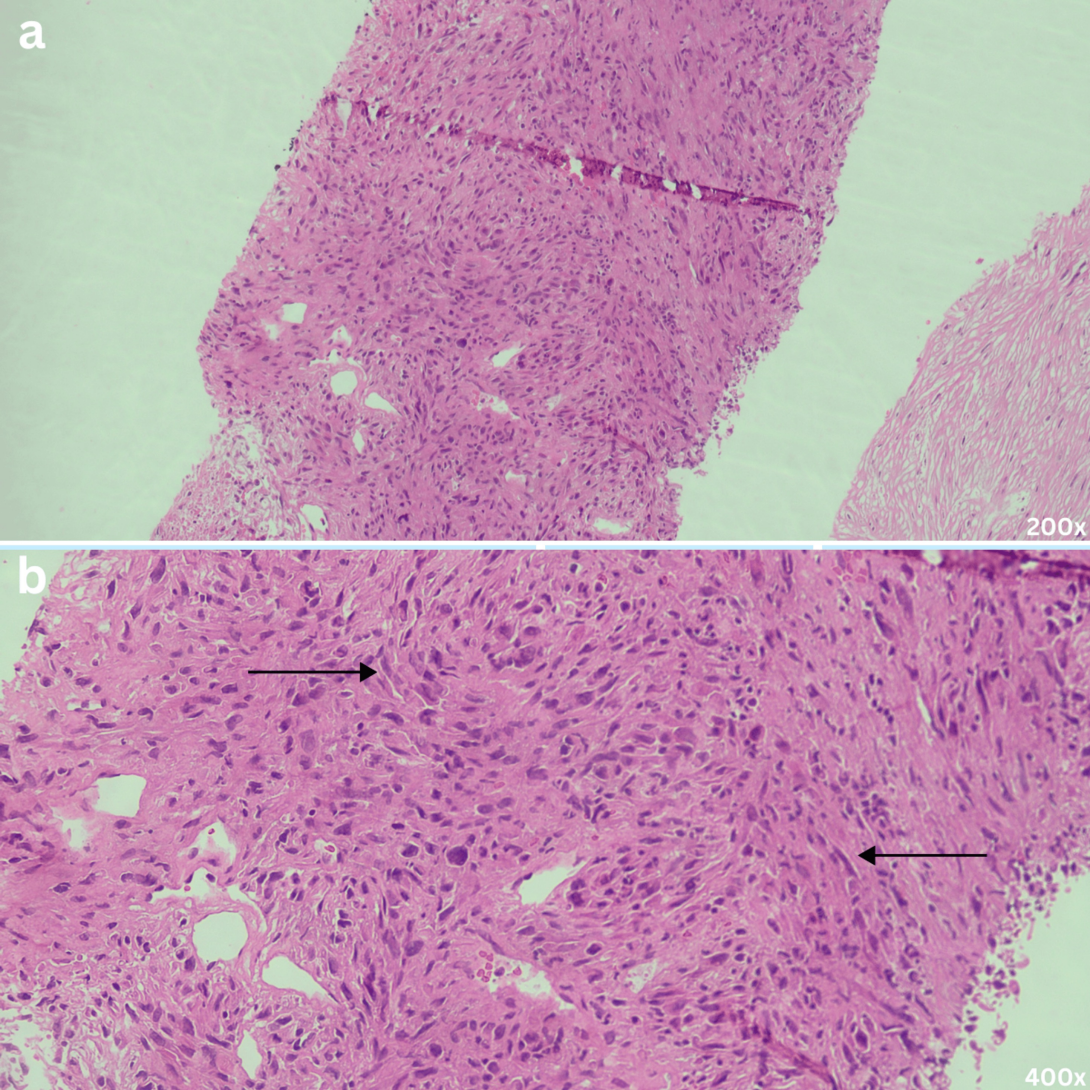
Histology Slides (a) Low-power (200x) and (b) high-power (400x) hematoxylin and eosin stained sections show an infiltrative neoplasm composed of atypical spindle cells (pointed by black arrows) with eosinophilic cytoplasm, pleomorphic hyperchromatic nuclei, and occasional mitotic figures, arranged in sheets with focal areas of necrosis.

Histopathological evaluation of the biopsy specimen revealed an infiltrating neoplasm composed of sheets of atypical spindle-shaped cells with areas of necrosis. The tumor cells exhibited eosinophilic cytoplasm and pleomorphic, oval, hyperchromatic nuclei, with occasional mitotic figures observed (Figure 2A-2B).

**Figure 3:**
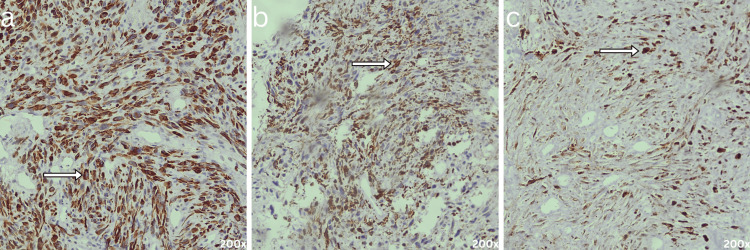
Immunochemistry staining (a) Pan-cytokeratin showing diffuse cytoplasmic positivity; (b) Glutamine synthetase demonstrating patchy cytoplasmic positivity; (c) HSP-70 exhibiting both cytoplasmic and nuclear positivity in tumor cells (indicated by arrowheads, at 200x magnification).

The diagnosis was further supported by immunohistochemistry, which showed positivity for two hepatocellular markers and negativity for markers of other lineages, including vascular, smooth muscle, adipose tissue, neural, and melanoma cells. The tumor cells were positive for cytokeratins (CK), confirming epithelial origin, and also expressed vimentin. Patchy positivity for glutamine synthetase and heat shock protein 70 (HSP-70) supported hepatocellular differentiation. This immunoprofile favored a diagnosis of SHCC (Figure 3A-3C).

Considering the patient’s clinical condition, significant functional decline, the extensive metastatic spread of the disease, and the unresectable nature of the tumor, surgical and locoregional treatments were deemed unsuitable. The patient was consequently referred to oncology, where a doxorubicin-based chemotherapy regimen was started, planned for 15 cycles at two-week intervals.

Following a brief initial improvement in his clinical condition, the patient’s health progressively declined. He was able to complete only nine cycles of doxorubicin before experiencing significant clinical deterioration. Despite supportive management, his condition continued to worsen, and he ultimately succumbed to the illness, passing away 5.5 months after the initial presentation.

## Discussion

SHCC is a rare malignancy characterized by the presence of both carcinomatous and sarcomatous components [10]. Although predominantly observed in older males, cases have also been documented in younger females [6,11]. The most common presentations include abdominal discomfort, weight loss, and fever [10,12]. Chronic viral hepatitis, particularly hepatitis C infection, has been reported to be associated with this rare tumor, with the risk increasing in the setting of cirrhosis due to ongoing hepatocellular injury and regeneration [5-7].

A notable feature of SHCC is the frequent observation of low or undetectable AFP levels, which can complicate early diagnosis and tumor monitoring. This is worth noting, as AFP is a commonly used tumor marker for assessing tumor burden and prognosis of HCC [4,7,9]. A recent retrospective study reported a median AFP level of 5.8 ng/mL in patients with SHCC. This study also highlighted that age below 55 years, Child-Pugh class B, and lower serum AFP levels were predictors of mortality [12].

Based on previous reports, SHCC predominantly involves the right lobe of the liver in approximately 90% of cases [9,10,12]. Common CT features include larger tumor size with peripheral rim-like or heterogeneous arterial enhancement, variable enhancement of the solid components with or without a tumor capsule, and evidence of intrahepatic metastases [9,10]. Other frequent radiologic findings include central tumor necrosis along with the tumor invasion into the adjacent organs and lymph node metastasis [12]. 

Diagnosis is confirmed on histopathological evaluation, with immunohistochemistry being essential; tumor cells typically express epithelial markers like cytokeratins K8/18 (and occasionally K7/19), and are negative for mesenchymal lineage markers, helping distinguish them from true sarcomas [6].

Unfortunately, treatment options for SHCC are limited due to the aggressive nature of the disease and its tendency to present at an advanced stage. In early stages, management may involve surgical resection and locoregional therapies; however, in advanced stages, systemic chemotherapy is typically employed. Despite these interventions, recurrence rates remain high, and overall outcomes are poor [4]. Recent evidence suggests that cyclin-dependent kinase 4 and 6 (CDK4/6) inhibitors, especially in cases with cyclin-dependent kinase inhibitor 2A and 2B (CDKN2A/B) loss, may represent a promising therapeutic strategy for SHCC, either alone or in combination with first-line agents like sorafenib or lenvatinib [13]. Additionally, tyrosine kinase inhibitors (TKIs) and immune checkpoint inhibitors (ICIs) have shown potential benefits, particularly in SHCC tumors that harbor CDKN2A mutations or express PD-L1 [14]. Despite encouraging responses in some cases, such as a reported instance where a 54-year-old man with advanced SHCC achieved an eight-month complete response with nivolumab, an anti-programmed cell death (PD-1) antibody, standardized treatment protocols and reliable predictive biomarkers are still lacking [15].

To date, only one case report has documented the use of doxorubicin combined with ifosfamide as systemic chemotherapy for recurrent SHCC after surgical resection, administered as salvage therapy. Acting via topoisomerase II inhibition, this regimen achieved over eight months of disease-free survival following seven cycles; however, there are no large-scale clinical trials to validate its efficacy [16]. Evidence supporting its role as palliative chemotherapy is extremely limited. In our case, doxorubicin was employed in a palliative setting, where data on its effectiveness remain scarce, and despite initial symptomatic improvement, the overall outcome was poor.

Patients with SHCC generally have a poorer prognosis compared to those with conventional HCC, with significantly shorter overall survival and lower recurrence-free survival rates. One study reported a poor one-year overall survival rate of 17.4% in patients with SHCC, while another documented a median overall survival of 8.3 months [17,4]. In contrast, patients who underwent liver resection had significantly better outcomes, with a one-year survival rate of 48.8% and a median survival of 10.5 months [12]. However, in our case report, overall survival was notably shorter at 5.5 months from initial presentation due to the presence of unresectable disease.

In summary, the aggressive nature of SHCC, combined with low AFP levels and variable imaging characteristics, makes early detection particularly difficult. Although a biopsy provides a definitive diagnosis, it is an invasive procedure and not always feasible for all patients. Histopathology with immunohistochemistry remains essential for accurate identification. This case underscores the aggressive clinical course and diagnostic challenges of SHCC, highlighting the urgent need for large-scale studies and the development of less invasive, more effective diagnostic tools and treatment strategies for high-risk individuals.

## Conclusions

SHCC is a rare and aggressive variant with nonspecific imaging findings and often normal AFP levels, making early diagnosis difficult. This case highlights the importance of high clinical suspicion in atypical clinical presentations, timely histopathological confirmation, and the limited benefit of conventional doxorubicin-based chemotherapy, underscoring the need for effective targeted therapies to improve its poor prognosis.
